# Dietary *Tribulus Terrestris* as a functional food combined with blood flow restriction to enhance the jump performance of basketball athletes: a randomized crossover study

**DOI:** 10.3389/fnut.2025.1648796

**Published:** 2025-08-13

**Authors:** Junhao Kong, Jinda Li, Mingchen Xu, Tao Liu, Zhiyu Xie, Tianhe Li, Yapu Liang

**Affiliations:** ^1^School of Sport Science, Beijing Sport University, Beijing, China; ^2^School of Journalism and Communication, Beijing Sport University, Beijing, China; ^3^School of Basic Medicine, Institute of Immunology, Tsinghua University, Beijing, China; ^4^Department of Physical Education, Peking University, Beijing, China; ^5^School of Strength and Conditioning Training, Beijing Sport University, Beijing, China

**Keywords:** *Tribulus terrestris*, blood flow restriction, post-activation potentiation, elite basketball players, performance

## Abstract

**Introduction:**

*Tribulus terrestris* (TT), a food-derived functional ingredient, may influence neuromuscular function via its bioactive compounds, but its acute effects on BFR-induced PAP and performance remain unknown. This study examined its short-term effects on neuromuscular performance after BFR-primed PAP in elite male basketball athletes (*n* = 20).

**Methods:**

Participants completed two sessions involving BFR-augmented plyometric protocols with either TT or placebo. CMJ performance was assessed at 0, 4-, 8-, 12-, and 16-min post-activation, measuring jump height (H(v)), peak/relative peak power (PP/RPP), maximum/relative force (MCF/MRF), peak rate of force development (PRFD), and modified reactive strength index (RSImod). Paired-sample *t*-tests (*p* < 0.05) were used for analysis.

**Results:**

In the placebo condition, H(v) increased at 4 and 8 min (*p* < 0.05), with PP and RPP peaking at 8 min (*p* < 0.05). TT supplementation enhanced early-phase force dynamics, elevating MCF (*p* = 0.057, 0 min; *p* < 0.01, 4 min) and PRFD (*p* = 0.002, 4 min), but attenuated H(v) (*p* < 0.001, 16 min), PP (*p* < 0.001), and RPP (*p* < 0.001) during later phases. Between-group comparisons revealed superior power metrics in the placebo group at 8–16 min (H(v): *p* = 0.001–0.017; RPP: *p* = 0.004–0.001), while TT transiently improved PRFD (*p* = 0.049, 0 min) and RSImod (*p* = 0.017, 4 min; *p* = 0.019, 16 min).

**Conclusion:**

Acute intake of TT modulates early-phase force responses but may impair sustained power output during PAP. Targeted timing and delivery formats should be considered in future food-first performance strategies.

## 1 Introduction

Optimal sports performance requires not only structured training but also tailored nutritional strategies that go beyond macronutrient provision. In recent years, the “food-first” paradigm has gained momentum, emphasizing the use of minimally processed, nutrient-dense whole foods and functional ingredients to support performance, recovery, and long-term athlete health (1). Functional foods rich in bioactive compounds such as polyphenols, flavonoids, and plant-derived saponins have demonstrated potential to modulate oxidative stress, inflammation, and neuromuscular readiness—key factors in high-performance environments (2, 3).

One such food-derived functional ingredient is *Tribulus terrestris* (TT), a flowering plant in the Zygophyllaceae family, has been widely used across traditional medicinal systems, particularly in South Asia, Central Asia, and the Mediterranean. The plant’s dried fruits, roots, and leaves are typically consumed in dietary forms such as powders, teas, and decoctions, or as part of fermented beverages and porridges (4, 5). In contemporary markets, TT is also processed into concentrated extracts and incorporated into encapsulated supplements, energy bars, and sports beverages (1, 4). Historically, it was marketed for the treatment of male hypogonadism and for its purported testosterone-enhancing and aphrodisiac effects (6). The bioactivity of TT has been attributed to its rich content of flavonoids, alkaloids, and steroidal saponins, particularly protodioscin, which are believed to modulate the endocrine and neuromuscular systems (5, 7). Preclinical studies in rodents have shown that supplementation with TT can increase plasma testosterone, stimulate IGF-1 signaling, promote skeletal muscle hypertrophy, and improve anaerobic performance (7–9). Mechanistically, these effects may be mediated by upregulation of anabolic pathways and modulation of the IGF-1/IGFBP axis (3, 10, 11). Additionally, TT exhibits antioxidant and anti-inflammatory properties, suggesting its potential to reduce muscle damage and support recovery in athletic populations (2, 12). However, evidence from human trials remains inconclusive: while some studies report no ergogenic or hormonal benefits in trained males (13, 14), others highlight context-specific physiological effects, particularly under stress or in synergy with other stimuli (5). These conflicting findings underscore the need to better understand the functional performance of TT in applied sports settings, especially when delivered through food-based or traditional formats.

Meanwhile, post-activation potentiation (PAP), a physiological phenomenon characterized by enhanced neuromuscular performance following prior muscle stimulation, has gained consensus in the field of sports science for its application in optimizing warm-up strategies (15, 16). Nevertheless, the mechanisms by which these effects are induced and the temporal characteristics of these phenomena remain subjects of ongoing academic debate. The preponderance of extant research suggests that near-maximal contractions (e.g., 80–130% 1RM) are requisite for the induction of PAP, with considerable enhancements in jump performance being observed 6 min following stimulation (17). The induction of PAP has also been demonstrated through explosive training or its combination with heavy resistance exercises (18). However, practical limitations, including equipment availability and the potential risks of injury associated with excessive loads, impede the implementation of these protocols in pre-competition settings. Inappropriate levels of intensity have been demonstrated to induce microstructural muscle damage, thereby attenuating the effects of PAP and compromising athletic performance (19). This challenge has prompted researchers to explore the potential of safer alternatives, such as blood flow restriction (BFR). Recent findings suggest that low-load BFR training (BFRT) can elicit comparable benefits to high-intensity physiological adaptations. This is achieved by accumulating metabolites and reducing blood flow, thereby offering a minimally invasive approach with potential benefits (15). In practice, BFR-induced PAP has demonstrated a distinctive capacity across various sports. For instance, the administration of lunge exercises with 130% arterial occlusion pressure (AOP) resulted in a significant enhancement of jump performance in trained males post-intervention (20). In a similar manner, the combination of BFR with whole-body vibration (WBV) and maximal voluntary contractions (MVC) led to an enhancement of PAP effects on vertical jump capacity (21). It has been demonstrated that standalone BFR interventions may not adequately induce PAP. However, when these interventions are paired with plyometric training, there is a notable synergistic effect, resulting in a substantial increase in countermovement jump (CMJ) and squat jump (SJ) heights within a time frame of 4–8 min (22). These findings offer novel insights into the optimization of warm-up strategies through the integration of insoles. While BFR-primed PAP protocols are increasingly used in elite sports, the potential synergy between acute functional food intake and such neuromuscular priming remains underexplored.

Therefore, the present study aimed to evaluate the acute effects of TT extract, administered in a food-relevant dosage and timing format, on BFR-induced PAP responses and vertical jump performance in elite male basketball players. By investigating both performance metrics and neuromuscular indicators across key time points post-intervention, this study provides insights into the feasibility of integrating TT as a functional food ingredient in pre-competition conditioning strategies.

## 2 Materials and methods

### 2.1 Participants

This study recruited 20 healthy male basketball athletes at the elite level or higher. The inclusion criteria were as follows: Male; Aged between 18 and 25 years; At least 3 years of regular basketball training experience (minimum of 2 sessions per week); Elite-level athletes or above; No history of arthritis, cardiovascular disease, or metabolic disorders. This threshold was set as a conservative lower limit to ensure eligibility, while still allowing for the possibility of variation due to training phase or competition calendar. In practice, all enrolled athletes reported a training frequency of at least 4 sessions per week, consistent with elite-level participation. No participants in this study trained fewer than three times per week. The exclusion criteria included: Musculoskeletal injuries or surgeries involving the lower limbs within the past 6 months; Presence of chronic pain or neuromuscular dysfunction; Use of any medications or supplements known to affect performance (e.g., creatine, caffeine) within the past 3 months; concurrent involvement in other sports-related intervention studies. A total of 20 male participants were enrolled in the study, and their basic characteristics are shown in Table 1.

**Table 1:** Participant characteristics.

Sample size	Age (years)	Height (cm)	Body mass (kg)
20	21.25 ± 1.77	177.25 ± 4.79	76.70 ± 6.17

The data are presented as M ± SD.

The study was conducted according to the guidelines of the Declaration of Helsinki and approved by the Sports Science Experiment Ethics Committee of Beijing Sport University (No. 2025134H). Written informed consent was obtained from all study participants.

### 2.2 Experimental design

This study utilized a randomized, counterbalanced crossover design involving two supplement intake conditions. In the first condition, participants consumed two capsules of TT extract (totaling 1,000 mg TT extract, 950 mg saponins; Viyouth, USA). In the second condition, participants took a placebo capsule, created by removing the original contents. The order of conditions was randomized by drawing sealed envelopes. Each session took place between 2–6 p.m. Each participant was asked to keep the start time of both sessions the same. Participants were not tested in a fasted state. However, all participants were from the same professional club and consumed standardized meals prepared under the supervision of a certified team nutritionist. Although individual dietary intake was not recorded, this controlled food environment ensured that nutritional status was comparable across test sessions. Furthermore, blinding procedures were applied to ensure that participants were unaware of the research hypothesis, and researchers remained blinded to the condition sequence until the start of each trial. This randomized crossover trial was designed and reported in accordance with the CONSORT 2010 guidelines (23), including the CONSORT extension for crossover trials (24).

### 2.3 Equipment and instrumentation

The device employed in this study was the SIEMENS Cypress PLUS (Siemens Medical Solutions USA, Inc.). Before the experiment began, participants’ lower extremity AOP at rest was measured using this device in combination with a pressurized belt equipped with a manometer. The blood flow restriction belt was obtained from Threatools, a company located in China. It was placed around the base of the participant’s thigh, at the level of the transverse gluteal muscle, and inflated to 50% of AOP as part of the PAP protocol. A rigorous data collection procedure was implemented using Kunwei force plates (Kunwei Sports Technology Corporation, China). These force plates have been thoroughly assessed for both reliability and validity, thereby ensuring the accuracy and integrity of the collected data (25).

### 2.4 Protocol and control

#### 2.4.1 Measurement of AOP

Two blood flow restriction belts are positioned at the base of the participant’s thighs, aligned with the transverse gluteal muscle. Simultaneously, the probe of the ultrasound Doppler device is coated with a coupling agent. An arterial flow signal is then located at the dorsalis pedis artery, indicated by either a pulsatile blood flow sound or visible waveforms. After a ten-minute period of supine rest, pressure is gradually increased until the blood flow signal disappears, followed by a slow release of pressure. The pressure value at which the Doppler probe detects the return of arterial blood flow is recorded as the AOP.

#### 2.4.2 Supplement intake

Subjects were allowed to ingest their respective capsules and given a 1-h rest period after randomization, during which no additional substances were ingested and no further exercise was performed.

#### 2.4.3 Warm-up and baseline measurement

The protocol commenced with a 5-min jog, followed by a standardized set of dynamic stretching exercises. This sequence included the knee–heel lift, goose balance, quadriceps stretch, and maximal stretch, each performed for six repetitions. After completing the warm-up, participants were instructed to perform two maximal-effort CMJ on a force platform. The data collected from these jumps were used as the baseline for subsequent analysis.

#### 2.4.4 Induction of PAP

Participants were equipped with blood flow restriction belts positioned at the base of the thighs and inflated to 50% of their AOP. They then performed two sets of ten straight-legged jumps, each set separated by a 30-s rest period and completed within 90 s. This was followed by three sets of five consecutive obstacle jumps, each at a height of 50 cm, with a 30-s rest between sets and a total duration of 120 s. Lastly, participants executed five drop jumps from a height of 50 cm, with 10-s intervals between jumps and a total completion time of 90 s. The entire sequence was completed within a five-minute period.

#### 2.4.5 Measurement of PAP effects

**Figure 1:**
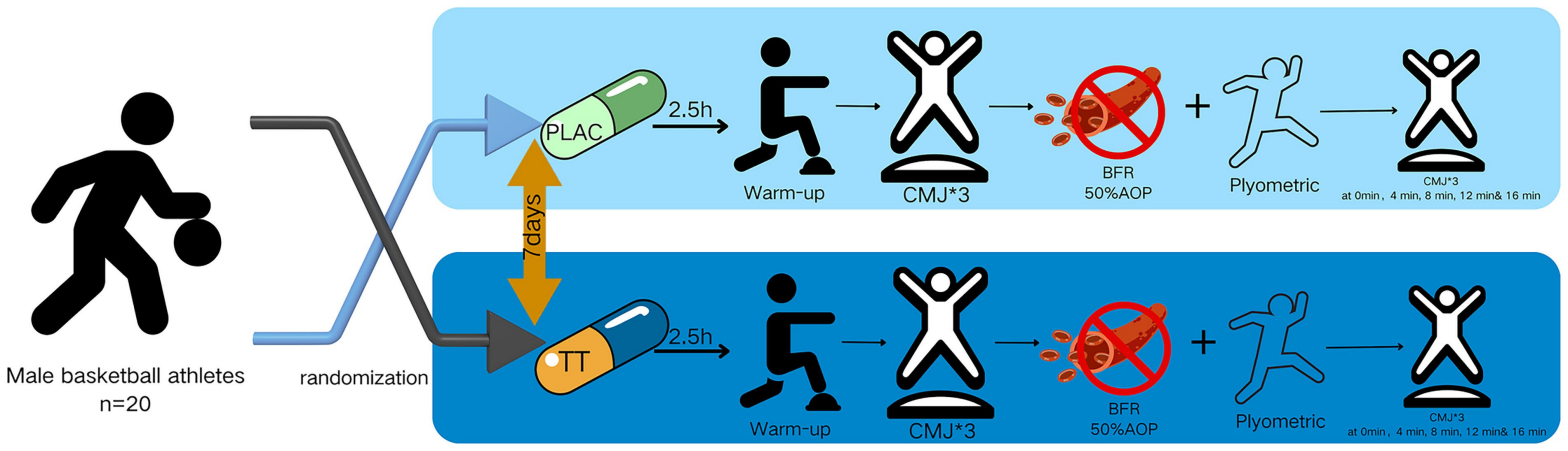
Flowchart of the experiment.

The temporal sequence of subsequent events occurred subsequent to the subjects’ completion of the plyometric training and the removal of the blood flow restriction belts. Two CMJ tests were then conducted at 0, 4, 8, 12, and 16 min (26, 27), and the data were recorded. The experimental procedure is illustrated in Figure 1.

### 2.5 Data collection and processing

Several parameters were calculated: velocity-calculated jump height (H(v), m), maximum combined force (MCF, N), maximum relative force (MRF, expressed in body weight), peak power (PP, W), relative peak power (RPP, W/kg), peak rate of force development (PRFD, N/s), and the modified reaction force index (RSImond). These metrics are automatically computed and stored by the KunWei force plates, which are integrated with the motor function performance test system software. The data must then be manually imported into Excel to facilitate organization and analysis. For each supplement intake condition, the percentage change of each metric at various time points relative to baseline values should be calculated.

### 2.6 Statistical analysis

All statistical analyses were performed using SPSS Version 27.0 (IBM Corp., Armonk, NY, USA), with significance set at *p* ≤ 0.05. The normality of all outcome variables was assessed using the Shapiro–Wilk test and further verified through visual inspection of Q–Q plots. Results are reported as mean ± standard deviation (SD). Paired-sample *t*-tests were used to compare each indicator at different time points within a specific supplement intake condition against baseline values. Additionally, paired-sample t-tests were applied to compare the percentage changes in each indicator at various time points between different supplement intake conditions. Effect sizes (Cohen’s *d*) were also calculated for all pairwise comparisons to quantify the magnitude of differences, interpreted according to conventional thresholds: small (*d* = 0.2), medium (*d* = 0.5), and large (*d* = 0.8).

## 3 Results

### 3.1 Effects of plyometric exercise with blood flow restriction on PAP following supplementation with placebo or *Tribulus terrestris*

The effects of BFR-augmented plyometric exercise on PAP induction under placebo or TT supplementation are summarized in Table 2. Placebo Condition Compared to baseline: H(v) significantly increased at 4 min (*p* = 0.013) and 8 min (*p* = 0.001). These improvements were accompanied by a medium effect size (*d* = 0.62 at 4 min) and a large effect size (*d* = 0.90 at 8 min), indicating substantial practical significance. PP (*p* = 0.007) and RPP (*p* = 0.012) showed significant improvements at 8 min. RSImod exhibited a notable decline at 16 min (*p* = 0.006).

**Table 2:** Effect of inducing PAP in the presence of placebo and *Tribulus terrestris* intake.

Variables	Time	Placebo			*Tribulus terrestris*		
		Mean ± SD	*p*	Cohen’s *d*	Mean ± SD	*p*	Cohen’s *d*
H(v) (m)	Baseline	0.45 ± 0.06	–	–	0.46 ± 0.06	–	–
	0 min	0.46 ± 0.07	0.355	0.21	0.46 ± 0.06	0.392	0.20
	4 min	0.48 ± 0.07	0.013	0.62	0.46 ± 0.06	0.145	0.34
	8 min	0.48 ± 0.08	0.001	0.90	0.45 ± 0.07	0.578	0.13
	12 min	0.47 ± 0.07	0.061	0.45	0.44 ± 0.07	<0.001	0.97
	16 min	0.46 ± 0.06	0.342	0.22	0.43 ± 0.06	<0.001	1.50
MCF (N)	Baseline	2036.10 ± 176.04	–	–	2017.57 ± 208.74	–	–
	0 min	2023.98 ± 196.28	0.666	0.10	2072.59 ± 257.77	0.057	0.45
	4 min	2109.01 ± 220.22	0.069	0.43	2160.41 ± 329.89	0.01	0.64
	8 min	2039.47 ± 196.47	0.926	0.02	2145.88 ± 396.77	0.064	0.44
	12 min	2042.11 ± 181.50	0.865	0.04	2110.91 ± 340.11	0.096	0.39
	16 min	2033.72 ± 190.08	0.946	0.02	2048.24 ± 215.31	0.365	0.21
MRF (BW)	Baseline	2.83 ± 0.22	–	–	2.76 ± 0.28	–	–
	0 min	2.84 ± 0.29	0.777	0.06	2.84 ± 0.34	0.034	0.51
	4 min	2.93 ± 0.27	0.057	0.45	2.95 ± 0.43	0.007	0.67
	8 min	2.84 ± 0.22	0.875	0.04	2.93 ± 0.52	0.063	0.44
	12 min	2.85 ± 0.22	0.594	0.12	2.88 ± 0.44	0.102	0.38
	16 min	2.83 ± 0.22	0.959	0.01	2.80 ± 0.29	0.373	0.20
PP (W)	Baseline	4542.59 ± 501.64	–	–	4576.39 ± 523.81	–	–
	0 min	4599.61 ± 519.49	0.313	0.23	4663.62 ± 466.35	0.068	0.43
	4 min	4627.42 ± 499.74	0.082	0.41	4697.64 ± 428.05	0.546	0.14
	8 min	4642.54 ± 510.94	0.007	0.67	4550.64 ± 490.77	0.28	0.25
	12 min	4582.57 ± 464.65	0.333	0.22	4447.48 ± 482.82	0.001	0.86
	16 min	4543.98 ± 480.77	0.972	0.01	4399.13 ± 461.73	<0.001	1.67
RPP (W/kg)	Baseline	61.79 ± 6.22	–	–	61.48 ± 6.17	–	–
	0 min	62.63 ± 6.90	0.327	0.23	62.83 ± 5.51	0.058	0.45
	4 min	63.02 ± 6.42	0.088	0.40	61.64 ± 4.61	0.78	0.06
	8 min	63.29 ± 6.66	0.012	0.62	61.03 ± 5.80	0.247	0.27
	12 min	62.37 ± 5.82	0.324	0.23	59.56 ± 5.45	0.001	0.91
	16 min	61.99 ± 5.96	0.746	0.07	59.00 ± 5.06	<0.001	1.38
PRFD (N/s)	Baseline	13081.47 ± 2908.64	–	–	12673.26 ± 3154.53	–	–
	0 min	12455.37 ± 3002.17	0.288	0.24	13575.56 ± 3044.68	0.1	0.39
	4 min	14686.96 ± 2715.59	0.097	0.39	15187.93 ± 3592.27	0.002	0.79
	8 min	13927.03 ± 2105.14	0.323	0.23	15100.19 ± 3420.88	0.007	0.68
	12 min	13560.72 ± 2583.26	0.606	0.12	14596.48 ± 3217.31	0.021	0.56
	16 min	13971.82 ± 1662.51	0.211	0.29	14171.46 ± 2295.95	0.019	0.58
RSImod	Baseline	0.72 ± 0.08	–	–	0.64 ± 0.08	–	–
	0 min	0.71 ± 0.11	0.280	0.25	0.63 ± 0.14	0.658	0.10
	4 min	0.68 ± 0.15	0.115	0.37	0.67 ± 0.12	0.097	0.39
	8 min	0.72 ± 0.12	0.812	0.05	0.66 ± 0.14	0.26	0.26
	12 min	0.68 ± 0.10	0.062	0.44	0.63 ± 0.14	0.912	0.03
	16 min	0.62 ± 0.12	0.006	0.69	0.63 ± 0.07	0.802	0.06

The data are presented as M ± SD. H(v), velocity-calculated jump height; MCF, maximum combined force; MRF, maximum relative force; PP, peak power; RPP, relative peak power; PRFD, peak rate of force development; RSImond, the modified reaction force index.

TT Supplementation Compared to baseline: H(v) decreased significantly at 16 min (*p* < 0.001). MCF demonstrated a trend toward improvement at 0 min (*p* = 0.057) showing a small effect size (*d* = 0.45) and significant increases at 4 min (*p* < 0.01) showing a medium effect (*d* = 0.64), underscoring the clinical relevance of early-phase force enhancement. While no statistical significance was observed at 8 min (*p* = 0.064) or 12 min (*p* = 0.096). MRF improved significantly at 0 min (*p* = 0.034) and 4 min (*p* < 0.007). PRFD increased consistently across multiple time points: 4 min (*p* = 0.002), 8 min (*p* = 0.007), 12 min (*p* = 0.021), and 16 min (*p* = 0.019). These changes were supported by medium effect sizes (*d* = 0.79 at 4 min; *d* = 0.68 at 8 min; *d* = 0.56 at 12 min; *d* = 0.58 at 16 min), confirming robust neuromuscular potentiation throughout the observation window. In contrast, PP and RPP declined markedly at 12 min (*p* = 0.001) and 16 min (*p* < 0.001).

### 3.2 Comparative analysis of PAP effects between placebo and *Tribulus terrestris* intake

**Figure 2:**
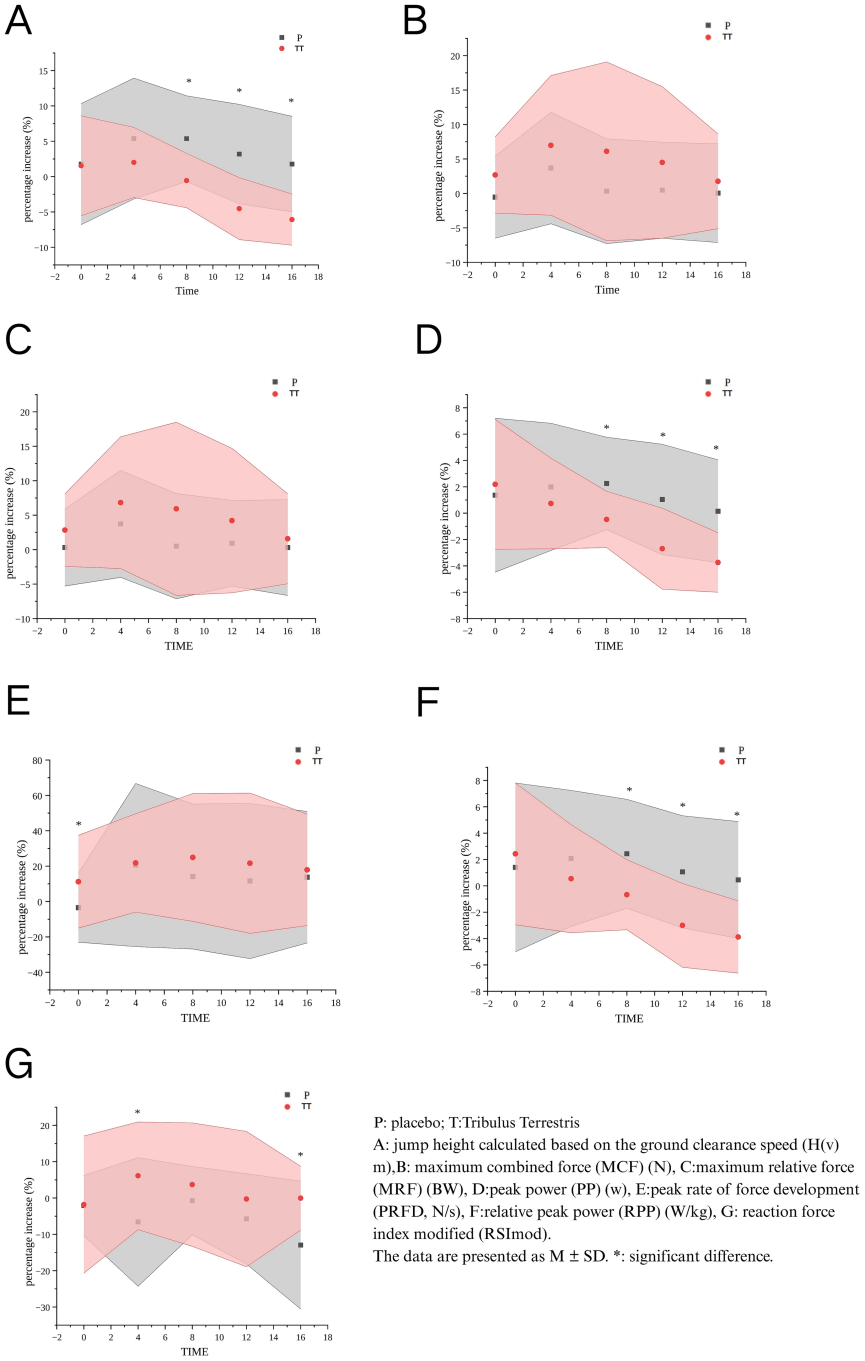
Comparative analysis of PAP effects between two conditions.

The between-group analysis of baseline-adjusted growth rates revealed the following outcomes: H(v): The placebo group demonstrated significantly higher values compared to the TT group at 8 min (*p* = 0.001), 12 min (*p* = 0.001), and 16 min (*p* < 0.001). Group differences at these time points exhibited large effect sizes (*d* > 0.90 for H(v) at 12–16 min), highlighting the magnitude of power output compromise under TT. PP: Placebo supplementation yielded superior performance over TT at 8 min (*p* = 0.003), 12 min (*p* = 0.008), and 16 min (*p* = 0.017). RPP: Significant advantages were observed in the placebo group at 8 min (*p* = 0.004), 12 min (*p* = 0.001), and 16 min (*p* < 0.001). PRFD: The TT group exhibited a transient advantage at baseline (0 min, *p* = 0.049). RSImod: TT supplementation showed intermittent improvements at 4 min (*p* = 0.019) and 16 min (*p* = 0.017). No significant intergroup differences were detected for MCF or MRF across all time points (Figure 2).

## 4 Discussion

The present study systematically investigated the acute effects of TT extract supplementation in conjunction with BFR-induced PAP on vertical jump performance in elite male basketball athletes.

The findings confirm that the combination of BFR and plyometric training elicits significant improvements in countermovement jump H(v) at 4- and 8-min post-intervention. Notably, PP and RPP also peaked at 8 min under the placebo condition, suggesting that the BFR-primed PAP protocol effectively enhances short-term neuromuscular output even in the absence of supplementation. These results are consistent with previous studies that identified a PAP time window ranging from 4 to 8 min following conditioning activities, during which maximal neuromuscular potentiation is typically observed (22). The decline in performance observed after 8 min further supports the transient nature of PAP, emphasizing the importance of timing when applying such interventions in pre-competition settings.

Under the placebo condition, the application of BFR-induced PAP produced performance enhancements that closely matched the established PAP time window. Specifically, H(v), PP, and RPP were significantly elevated at 8 min post-conditioning. These findings align with previous work showing that PAP typically peaks between 4–8 min following conditioning activity, particularly when paired with plyometric or resistance-based stimulation (20, 26). The improvement in vertical jump performance observed in the placebo condition may be attributed to increased motor unit recruitment, optimized muscle-tendon synchronization, and elevated phosphorylation of myosin regulatory light chains—mechanisms that transiently enhance muscle contraction speed and force output (17, 27). This outcome confirms that BFR, even at submaximal intensities, can acutely amplify neuromuscular readiness in elite athletes, particularly during the 4–8 min post-activation window (21).

In contrast, the TT condition displayed a distinct performance profile. While TT supplementation did not enhance H(v), PP, or RPP at any timepoint, it did result in significant improvements in MCF, MRF, and PRFD, particularly during the early phase (0–4 min). This suggests a possible early enhancement in force-generating capacity, without a corresponding improvement in explosive movement outcomes. Notably, PRFD remained elevated from 4 through 16 min, and RSImod—a composite indicator of reactivity and neuromuscular efficiency—was improved at both 4 and 16 min. These effects may be explained by TT’s potential to modulate IGF-1 and GH pathways, improve calcium handling at the neuromuscular junction, and reduce exercise-induced oxidative stress (3, 10, 28). However, the absence of improvements in power and jump height suggests that force enhancements alone may be insufficient for producing complex athletic movements, which depend on rapid force application, inter-muscular coordination, and velocity transfer.

When comparing the two conditions, a clear trade-off pattern emerges. The placebo condition induced a classic PAP response—enhanced mechanical power and vertical displacement during the 8- to 16-min window. Conversely, TT supplementation shifted the neuromuscular profile toward a more force-dominant state, characterized by early-phase gains in MCF, MRF, and PRFD, but accompanied by compromised peak power output and jump height. This shift was evidenced by a medium effect size for early-phase PRFD (*d* = 0.79) and a medium effect size for MCF (*d* = 0.64), suggesting that TT’s force-enhancing effects are physiologically meaningful despite attenuated power metrics.

This performance dissociation could be attributed to neuromechanical interference, such as altered stretch-shortening cycle dynamics, central nervous system fatigue, or delayed excitation-contraction coupling, potentially influenced by the bioactive compounds in TT. One plausible explanation is that certain bioactive compounds in *Tribulus terrestris*, particularly protodioscin, may acutely stimulate endocrine pathways, such as by increasing luteinizing hormone (LH) or modulating the hypothalamic–pituitary axis (5). However, these same compounds may also exert less favorable effects on the neuromechanical coordination required for explosive performance. For instance, alterations in neurotransmitter balance—particularly involving serotonin or dopamine (29)—or disruptions in calcium handling at the neuromuscular junction (30) may delay muscle relaxation or impair timing-dependent motor control. These effects could interfere with rapid force transmission or stretch-shortening cycle efficiency, which are essential for achieving high jump height or peak power output. Supporting this, Rogerson et al. (8) reported no improvements in explosive performance among elite athletes supplemented with TT, suggesting a mismatch between its acute physiological effects and the demands of high-speed athletic tasks. Furthermore, animal studies have suggested that while TT may promote muscle hypertrophy and recovery over the long term, its acute ergogenic effects are inconsistent and may not align with short PAP timeframes (7, 8).

Overall, these findings underscore the complexity of integrating functional food-derived compounds into pre-competition conditioning. While TT may acutely enhance parameters linked to strength and reactivity, its interference with power-dependent outcomes—critical in sports like basketball—suggests that timing, dosing, and formulation (e.g., extract vs. whole food) must be carefully considered to avoid counterproductive effects. Further research using electromyography, hormonal profiling, and time-matched metabolic measures is warranted to elucidate the underlying interaction mechanisms and optimize application protocols.

No adverse effects or discomfort were reported by any participants throughout the course of this study, indicating good short-term tolerability of the administered *Tribulus terrestris* dosage in a food-based format. This is consistent with several human studies that also found TT to be generally safe when used within recommended dosages (13). However, previous reports—particularly in animal models and high-dose supplementation trials—have suggested potential side effects such as hepatotoxicity, nephrotoxicity, or hormonal imbalances when administered chronically or at pharmacological concentrations (31). While these effects were not observed in the present protocol, practitioners should remain cautious regarding prolonged or excessive intake, especially in unregulated supplement forms.

From a practical nutrition standpoint, the findings of this study raise important considerations for the use of TT as a functional food ingredient in athletic settings. Although TT did not enhance jump height or power output during the typical PAP peak window, its capacity to acutely improve force-related metrics such as PRFD and RSImod suggests that it may have specific value in enhancing neuromuscular responsiveness and preparatory muscle tension. These characteristics are particularly relevant in explosive sports like basketball, where brief periods of high-reactivity movement—such as cutting, rebounding, and first-step acceleration—play a crucial role in game success.

Given its traditional use as a food component, TT holds promise for integration into pre-competition meals, natural performance drinks, or functional snacks. Traditional delivery forms—such as TT-infused herbal teas, powdered fruit mixed with yogurt or porridge, and fermented beverages—could offer athletes a more physiologically harmonious alternative to concentrated capsules. The use of such formats also aligns with the food-first paradigm in sports nutrition, which prioritizes whole-food solutions to enhance bioavailability, reduce side effects, and support long-term athlete health (1, 4).

However, the timing and dosage of TT ingestion remain critical. The observed reduction in jump-related power output during the 8–16 min window in this study indicates that certain phytochemical effects may transiently disrupt neuromuscular coordination or recovery processes if consumed too close to performance. Therefore, TT-containing functional foods may be more suitable when consumed earlier in the warm-up routine—targeting early-phase potentiation—or in training blocks where reactive strength is prioritized over peak power output. In addition, individual variability in metabolic response, hormonal sensitivity, and exercise context must be accounted for when designing TT-enriched nutrition plans.

Looking ahead, future studies should explore the ergogenic potential of whole-food forms of TT (e.g., standardized tea, powdered fruit blends) compared to isolated extracts, with a focus on delivery matrix, absorption kinetics, and synergy with other plant bioactives. Investigations should also assess long-term effects on training adaptation, muscle recovery, and hormonal regulation. By integrating TT into sport-specific nutrition strategies, practitioners may unlock new pathways to enhance explosive readiness while maintaining a food-first, athlete-centered nutritional philosophy.

This study is not without limitations. First, the relatively small sample size (*n* = 20) may reduce statistical power and limit the generalizability of the findings, increasing the potential for Type II errors. Second, the homogeneity of the sample—comprising only elite-level male basketball athletes—restricts the applicability of results to female athletes, youth players, or recreational populations with different neuromuscular and metabolic profiles. Third, both the blood flow restriction pressure (50% of arterial occlusion pressure) and the standardized dosage of TT extract may not reflect optimal thresholds for all individuals, as athlete-specific responses to BFR and phytochemical interventions are known to vary. Finally, a temporal mismatch was observed between the PAP response window (with performance peaking at 4–8 min post-conditioning) and the apparent performance decline following TT ingestion during the same period. This suggests that timing of functional food ingestion is critical, and that the ergogenic potential of TT may depend on phase-specific alignment with neuromuscular readiness. Future studies should investigate individualized timing strategies and explore different delivery formats—including whole-food preparations—tailored to the specific temporal demands of training or competition. By refining such parameters, researchers and practitioners can better harness the potential of functional food components within precision-based, food-first performance frameworks.

## 5 Conclusion

In summary, the consumption of TT in encapsulated form shows promise in modulating neuromuscular readiness when paired with blood flow restriction protocols. While our findings indicate short-term enhancements in force-related parameters, they also suggest potential compromises in sustained power output. These effects underscore the need for nuanced application in pre-competition strategies. From a food-first perspective, the incorporation of TT into functional sports foods—such as herbal infusions, natural performance snacks, or meal-based formulations—merits further investigation. Future studies should examine not only the efficacy of these forms but also their bioavailability, safety, and acceptability in elite athletic populations.

## Data Availability

The original contributions presented in the study are included in the article/supplementary material, further inquiries can be directed to the corresponding authors.
